# Machine Learning‐Based Prediction of Brain Metastasis at Initial Diagnosis in Small‐Cell Lung Cancer: Model Development and SHAP Interpretation Study

**DOI:** 10.1002/cnr2.70625

**Published:** 2026-07-16

**Authors:** Yu‐Long He, Qin‐Ling Jiang, Yong Zhai, Xian‐Ling Zhao, Zi‐Kun Huang

**Affiliations:** ^1^ Department of Oncology Nanxishan Hospital of the Guangxi Zhuang Autonomous Region (The Second People's Hospital of Guangxi Zhuang Autonomous Region) Guilin Guangxi China; ^2^ College of Information Engineering Guilin University Guilin Guangxi China

**Keywords:** brain metastasis, machine learning, prediction model, SHapley additive exPlanations, small‐cell lung cancer

## Abstract

**Background:**

Brain metastasis (BM) in small cell lung cancer (SCLC) is typically associated with poor survival rates and quality of life, making the timely identification of patients with a high likelihood of BM at diagnosis crucial.

**Aims:**

To develop and validate a machine learning (ML) prediction model for BM in the overall SCLC population and provide an interpretable and clinically accessible risk assessment tool.

**Methods and Results:**

Univariate and multivariate logistic regression analyses were performed to identify BM‐associated factors. Eight ML algorithms were applied to build the model. The model performance was quantified using the area under the curve (AUC), area under the precision–recall curve (AUPRC), and Matthews correlation coefficient (MCC). SHapley Additive exPlanations (SHAP) was used to interpret the best‐performing model. A web calculator was developed to facilitate individualized BM risk estimation. Multivariate logistic regression revealed that age, T stage, tumor size, bone metastasis, lung metastasis, and distant lymph node metastasis were independently associated with BM. Extreme Gradient Boosting (XGB) achieved the best discrimination in the validation cohort, with an AUC of 0.8762, AUPRC of 0.9025, accuracy of 0.7974, precision of 0.8009, recall of 0.7974, specificity of 0.8516, MCC of 0.5983, F1‐score of 0.7968, and Brier score of 0.1377. Cross‐validation demonstrated similarly strong performance. SHAP analysis identified age, tumor size, T stage, distant lymph node metastasis, bone metastasis, and lung metastasis as the strongest contributors to BM risk. A web‐based risk calculator was developed to facilitate exploratory risk stratification and individualized BM risk estimation.

**Conclusion:**

We created and internally validated an interpretable ML model to identify brain metastasis at diagnosis in SCLC, with XGB showing the best performance. Its web‐based tool may assist in identifying patients with a higher likelihood of brain metastasis at diagnosis and provide supplementary risk stratification information for exploratory clinical assessment. Further prospective validation is required before routine clinical implementation of this model.

## 1 Introduction

Small‐cell lung cancer (SCLC) is a highly heterogeneous and profoundly aggressive pulmonary neuroendocrine tumor characterized by rapid growth and a strong tendency for early distant dissemination. Brain metastasis (BM) is one of the most common metastatic sites in SCLC, occurring in approximately 16.4% of patients at initial diagnosis and rising to 40%–50% during the disease course; among patients who survive beyond 2 years, the cumulative incidence can reach 60%–80% [1]. BM development markedly worsens neurological function, quality of life, and survival. For extensive‐stage SCLC (ES‐SCLC), which frequently includes patients with BM, the 5‐year survival rate remains only 1%–2%, highlighting the lethality of this disease and the persistent lack of effective prognostic tools [2].

Prophylactic cranial irradiation (PCI) can reduce the incidence of BM by approximately 50%. However, contemporary evidence indicates that PCI does not improve overall survival (OS) and may contribute to neurocognitive decline [3]. The routine use of PCI has become increasingly controversial in the era of magnetic resonance imaging (MRI). Current international guidelines recommend PCI selectively, primarily for patients with limited‐stage SCLC (LS‐SCLC) who respond to initial therapy. Despite advances in multimodal treatment, including surgery, radiotherapy, chemotherapy, targeted therapy, immunotherapy, and optimized supportive care, the overall therapeutic outcomes for SCLC remain limited, with a median OS of approximately 5 months in many settings [4, 5, 6]. Consequently, the accurate identification of patients with a high likelihood of brain metastasis at diagnosis may support individualized imaging evaluation and clinical risk stratification in patients with SCLC.

Although several studies have explored the risk factors for BM in SCLC, existing predictive tools remain scarce and have a limited scope. Many rely on traditional regression approaches, are restricted to extensive‐stage disease, or lack external validations. Therefore, a robust, accurate, and clinically applicable prediction model capable of identifying high‐risk individuals across the entire SCLC population is urgently required. Such a model could facilitate proactive surveillance, support exploratory risk stratification and individualized imaging assessment, and ultimately improve clinical outcomes [7].

Machine learning (ML), a branch of artificial intelligence (AI), offers substantial advantages in clinical prediction. By capturing complex, nonlinear interactions among variables, ML methods frequently outperform traditional statistical models in forecasting cancer progression and metastasis. ML‐based approaches have been widely applied to support personalized treatment recommendations, improve diagnostic accuracy, and enhance healthcare efficiency [8, 9, 10].

To address the existing clinical gap, this study utilized the population‐based Surveillance, Epidemiology, and End Results (SEER) database to systematically examine the risk factors associated with BM in SCLC. We developed and compared multiple ML algorithms to construct an accurate and interpretable BM prediction model for the entire SCLC cohort. Furthermore, we built a web‐based risk calculator to facilitate accessibility and exploratory applications in making informed decisions to minimize or delay the development of BM.

## 2 Materials and Methods

### 2.1 Study Design and TRIPOD‐ML Compliance

This study followed the Transparent Reporting of a Multivariable Prediction Model for Individual Prognosis or Diagnosis—Machine Learning extension (TRIPOD‐ML) guidelines for the development and validation of supervised ML prediction models. This study aimed to develop a prognostic model for the occurrence of brain metastasis at diagnosis in patients with SCLC.

### 2.2 Data Source and Study Population

Data were obtained from the SEER Incidence Research Plus database (17 registries; November 2023 submission) [11]. Patients diagnosed with SCLC between 2010 and 2021 were included, as detailed information on metastases to the liver, brain, bone, and lung became consistently available in SEER in 2010 [12]. Data extraction was performed using SEER*Stat version 8.4.4.

Eligible patients were those with histologically confirmed SCLC, defined using the International Classification of Diseases for Oncology (ICD‐O‐3) histology code 8041/3 [13], a clearly documented distant metastasis status, and age ≥ 25 years at diagnosis. Cases identified only through autopsies or death certificates were excluded. Additional exclusion criteria included a history of another primary malignancy, unknown or inapplicable TN staging (T0, Tis, or missing), missing racial information, missing tumor size, and incomplete clinical records. As SEER program provides publicly available de‐identified data, this study did not require institutional review board approval or patient consent.

In the external validation cohort, 485 patients were recruited from Nanxishan Hospital of Guangxi Zhuang Autonomous Region, following the aforementioned inclusion and exclusion criteria.

Imaging examinations for initial cancer diagnosis and distant metastasis screening (including BM assessment) were conducted synchronously at the time of initial pathological diagnosis for all enrolled patients. All included cases adopted a unified diagnostic workflow: histological confirmation of SCLC and systemic metastasis screening via imaging were completed within the same diagnostic time window, so no temporal interval existed between cancer diagnosis and metastasis imaging screening across the entire study cohort, and imaging timing was fully standardized for all eligible cases.

Notably, the SEER database only documents the metastatic status confirmed at the time of initial SCLC diagnosis. Accordingly, the prediction model developed in this study is designed to identify synchronous BM present at initial diagnosis, rather than forecast the risk of de novo BM that develops during long‐term follow‐up after diagnosis.

### 2.3 Data Processing and Variable Definition

Staging information was harmonized according to the 8th edition of the American Joint Committee on Cancer (AJCC) TNM classification following the SEER guidelines. The variable definitions and categorizations used for model construction are summarized in Tables S1 and S2. Metastases to the contralateral lung are categorized as lung metastasis in this study. Multiple tumor nodules in the ipsilateral lung are defined as an independent variable and not counted as lung metastasis.

**FIGURE 1:**
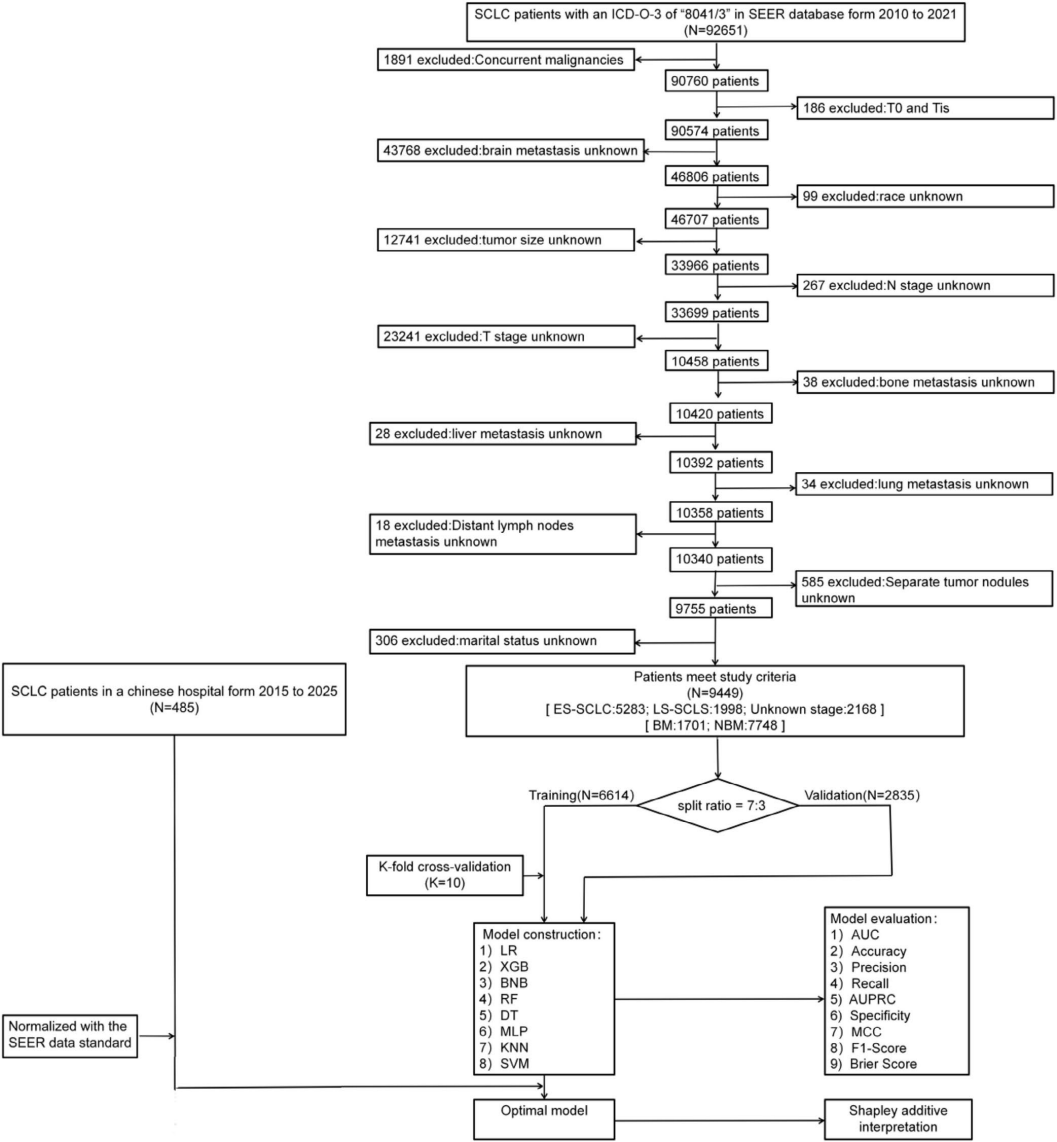
Patient selection flowchart and technical route. AUC, area under the curve; AUPRC, area under the precision‐recall curve; BM, brain metastasis; BNB, Bernoulli Naïve Bayes; DT, decision tree; ES‐SCLC, extensive‐stage SCLC; KNN, K‐nearest neighbor; LR, logistic regression; LS‐SCLC, limited‐stage SCLC; MCC, Matthews correlation coefficient; MLP, multilayer perceptron; NBM, no brain metastasis; RF, random forest; SCLC, small cell lung cancer; SVM, support vector machine; XGB, extreme gradient boosting machine.

Patients with incomplete information on any required variable were excluded according to the predefined eligibility criteria; therefore, no data imputation was performed. Because a complete case analysis approach was used, the potential for selection bias cannot be entirely excluded. After applying all selection steps, a final cohort of 9449 eligible patients with SCLC was identified, including 1998 patients with limited‐stage disease, 5283 with extensive‐stage disease, and 2168 with unknown stage. The patient selection process is shown in Figure 1.

### 2.4 Predictor Selection

Thirteen clinically relevant demographic and tumor‐related variables were considered. As the objective was to identify brain metastasis at diagnosis, the BM variable was removed from the candidate predictors. The remaining 12 variables (age, sex, race, marital status, T stage, N stage, tumor size, separate tumor nodules in the ipsilateral lung, bone metastasis, liver metastasis, lung metastasis, and distant lymph node metastasis) were retained. These variables were selected based on their consistent availability in the SEER database, established biological or clinical relevance, and suitability for ML algorithms, which can accommodate complex interactions and non‐linear effects without requiring restrictive feature selection.

### 2.5 Model Development

The study cohort was randomly divided into training (70%) and validation (30%) sets. Eight supervised machine‐learning algorithms have been developed: Logistic Regression (LR) [14], Extreme Gradient Boosting (XGB) [15], Bernoulli Naïve Bayes (BNB) [16], Random Forest (RF) [17], Decision Tree (DT) [18], Multilayer Perceptron (MLP) [19], K‐Nearest Neighbor (KNN) [20], and Support Vector Machine (SVM) [21]. All models were trained using repeated 10‐fold cross‐validation with 50 iterations performed using different random seeds to reduce variance and ensure robust performance estimates [22].

As only 18% of patients had BM, class imbalance was assessed. Preliminary analyses showed that oversampling techniques (e.g., SMOTE) and class‐weighted algorithms did not meaningfully improve model discrimination or calibration. Therefore, all models were trained on the original dataset without rebalancing, and repeated cross‐validation was used to mitigate the effects of class imbalance.

The hyperparameters for all algorithms were tuned using a random search strategy, which allowed efficient exploration of the parameter space while reducing the risk of overfitting. The optimal hyperparameter sets identified through cross‐validation were used to fit the final models.

Additionally, models were validated using data from 485 SCLC patients from a Chinese hospital to evaluate the model performance and generalizability.

### 2.6 Model Evaluation and Interpretability

The model performance was evaluated using nine metrics: area under the receiver operating characteristic curve (AUC), accuracy, precision, recall, area under the precision–recall curve (AUPRC), specificity, Matthews correlation coefficient (MCC), F1‐score, and Brier score [8]. Because the dataset was imbalanced, AUPRC was emphasized as a more reliable measure of discriminative ability than AUC [23]. Additional metrics, including MCC and Brier score, were also evaluated to provide a more comprehensive assessment of model performance under imbalanced conditions. Receiver operating characteristic curves, precision–recall curves, calibration curves, and decision curve analyses (DCA) were generated to assess discrimination, calibration, and clinical utility.

The best‐performing model was further examined using SHapley Additive exPlanations (SHAP), which provide both global and local interpretability of ML predictions. SHAP values were used to quantify the contribution of each predictor to the individual and overall model outputs, thereby enhancing the clinical interpretability of the final model [24].

### 2.7 Statistical Analysis

All statistical analyses were conducted using Python version 3.8 and R version 4.1.0 software. Continuous variables are summarized as medians with interquartile ranges (IQRs) and were compared using the Wilcoxon rank‐sum test. Categorical variables are described as frequencies and percentages and compared using the chi‐squared test or Fisher's exact test, as appropriate. Univariate logistic regression was used to identify variables associated with BM, and variables with *p* < 0.05 were entered into multivariate logistic regression to determine independent risk factors for BM. Categorical variables were analyzed using predefined reference categories, and odds ratios (ORs) with 95% confidence intervals (CIs) were reported for all logistic regression analyses. These regression analyses were used for clinical interpretation rather than restricting the ML models, which included all selected predictors.

## 3 Results

### 3.1 Demographic and Clinical Characteristics

A total of 9449 patients with SCLC diagnosed between 2010 and 2021 were included in the study. Among them, 1701 (18%) had brain metastasis at diagnosis and 7748 (82%) did not. The baseline demographic and clinical characteristics of the patients are summarized in Table 1. Patients were randomly divided into training (*n* = 6614) and validation (*n* = 2835) sets in a 7:3 ratio. The characteristics of the two datasets are presented in Table 2.

**TABLE 1:** Clinical characteristics of study population.

Variables	All	BM	NBM	*p* value
	*N* = 9449	*N* = 1701	*N* = 7748	
Age (mean (SD))	67.58 (8.94)	66.31 (9.04)	68.00 (9.05)	< 0.001*
Gender, *n* (%)				0.292
Male	4532 (47.96)	836 (49.15)	3696 (47.70)	
Female	4917 (52.04)	865 (50.85)	4052 (52.30)	
Race, *n* (%)				0.059
White	8186 (86.63)	1451 (85.30)	6735 (86.93)	
Black	822 (8.70)	173 (10.17)	649 (8.38)	
Other	441 (4.67)	77 (4.630)	364 (4.70)	
Marital status, *n* (%)				0.027*
Divorced	134 (1.42)	27 (1.59)	107 (1.38)	
Married (including common law)	4536 (48.01)	832 (48.91)	3704 (47.81)	
Separated	1553 (16.44)	237 (13.93)	1316 (16.99)	
Single (never married)	1493 (15.80)	262 (15.40)	1231 (15.89)	
Unmarried or domestic partner	64 (0.68)	13 (0.76)	51 (0.66)	
Widowed	1669 (17.66)	330 (19.40)	1339 (17.28)	
T stage, *n* (%)				< 0.001*
T1	1902 (20.13)	272 (15.99)	1630 (21.04)	
T2	1788 (18.92)	323 (18.99)	1465 (18.91)	
T3	1813 (19.19)	326 (19.17)	1487 (19.19)	
T4	3946 (41.76)	780 (45.86)	3166 (40.86)	
N stage, *n* (%)				0.001*
N0	1664 (17.61)	265 (15.58)	1399 (18.06)	
N1	861 (9.11)	136 (8.00)	725 (9.36)	
N2	4548 (48.13)	815 (47.91)	3733 (48.18)	
N3	2376 (25.15)	485 (28.51)	1891 (24.41)	
Tumor size (mean (SD)) (mm)	51.94 (30.08)	53.38 (29.90)	51.63 (30.12)	0.013*
Separate tumor nodules ipsilateral lung, *n* (%)				0.001*
None	7083 (74.96)	1213 (71.31)	5870 (75.76)	
Same lobe	865 (9.15)	171 (10.05)	694 (8.96)	
Different lobe	508 (5.38)	95 (5.58)	413 (5.33)	
Same and different lobes	356 (3.77)	75 (4.41)	281 (3.63)	
Unknown if same or different lobe	637 (6.74)	147 (8.64)	490 (6.32)	
Mets at DX‐bone, *n* (%)				< 0.001*
No	7070 (74.82)	1176 (69.14)	5894 (76.07)	
Yes	2379 (25.18)	525 (30.86)	1854 (23.93)	
Mets at DX‐liver, *n* (%)				0.001*
No	6660 (70.48)	1140 (67.02)	5520 (71.24)	
Yes	2789 (29.52)	561 (32.98)	2228 (28.76)	
Mets at DX‐lung, *n* (%)				< 0.001*
No	8421 (89.12)	1445 (84.95)	6976 (90.04)	
Yes	1028 (10.88)	256 (15.05)	772 (9.96)	
Mets at DX‐Distant LN, *n* (%)				< 0.001*
No	8174 (86.51)	1337 (78.60)	6837 (88.24)	
Yes	1275 (13.49)	364 (21.40)	911 (11.76)	

*Note:* Other race: American Indian/AK Native, Asian/Pacific Islander.

Abbreviations: At DX, at the time of diagnosis; BM, brain metastasis; LN, lymph nodes; Mets, metastasis; mm, millimeter; NBM, no brain metastasis.

* *p* < 0.05.

**TABLE 2:** Clinical characteristics of the training and validation set.

Variables	SEER database (*N* = 9449)		*p* value
	Training (*N* = 6614)	Validation (*N* = 2835)	
Age (mean (SD))	67.83 (9.08)	67.40 (9.04)	0.020*
Gender, *n* (%)			
Male	3167 (47.88)	1365 (48.05)	0.822
Female	3447 (52.12)	1470 (51.85)	
Race, *n* (%)			
White	5728 (86.60)	2458 (86.70)	0.075
Black	559 (8.45)	263 (9.28)	
Other	327 (4.94)	114 (4.02)	
Marital status, *n* (%)			0.037
Divorced	1025 (15.50)	468 (16.51)	
Married (including common law)	3205 (48.46)	1331 (46.95)	
Separated	103 (1.56)	31 (1.09)	
Single (never married)	1133 (17.13)	536 (18.91)	
Unmarried or domestic partner	40 (0.60)	24 (0.85)	
Widowed	1108 (16.75)	445 (15.70)	
T stage, *n* (%)			0.211
T1	1297 (19.61)	605 (21.34)	
T2	1264 (19.11)	524 (18.48)	
T3	1290 (19.50)	523 (18.45)	
T4	2763 (41.78)	1183 (41.73)	
N stage, *n* (%)			0.245
N0	1185 (17.92)	479 (16.90)	
N1	621 (9.39)	240 (8.47)	
N2	3164 (47.84)	1384 (48.82)	
N3	1644 (24.86)	732 (25.82)	
Tumor size (mean (SD)) (mm)	52.05 (29.98)	61.69 (30.32)	0.411
Separate tumor nodules ipsilateral lung, *n* (%)			0.430
None	4923 (74.43)	2160 (76.19)	
Same lobe	624 (9.43)	241 (8.50)	
Different lobe	365 (5.52)	143 (5.04)	
Same and different lobes	250 (3.78)	106 (3.74)	
Unknown if same or different lobe	452 (6.83)	185 (6.53)	
Mets at DX‐bone, *n* (%)			0.134
No	4978 (75.26)	2092 (73.79)	
Yes	1636 (24.74)	743 (26.21)	
Mets at DX‐liver, *n* (%)			0.749
No	4655 (70.38)	2005 (70.72)	
Yes	1959 (29.62)	830 (29.28)	
Mets at DX‐lung, *n* (%)			0.130
No	5873 (88.80)	2548 (89.88)	
Yes	741 (11.20)	287 (10.12)	
Mets at DX‐Distant LN, *n* (%)			0.669
No	5728 (86.60)	3446 (86.28)	
Yes	886 (13.40)	389 (13.72)	
BM, *n* (%)			1
No	5423 (81.99)	2325 (82.01)	
Yes	1191 (18.01)	510 (17.99)	

*Note:* Other race: American Indian/AK Native, Asian/Pacific Islander.

Abbreviations: At DX, at the time of diagnosis; BM: brain metastasis; LN, lymph nodes; Mets, metastasis; mm, millimeter.

* *p* < 0.05.

### 3.2 Logistic Regression Analysis

Univariate logistic regression identified several variables significantly associated with BM in the training cohort, including age, T stage, N stage, tumor size, separate tumor nodules in the ipsilateral lung, bone metastasis, liver metastasis, lung metastasis, and distant lymph node metastasis (*p* < 0.05) (Table 3). Multivariate logistic regression showed that age (*p* < 0.000), T stage (*p* < 0.05), tumor size (*p* = 0.046), bone metastasis (*p* = 0.001), lung metastasis (*p* = 0.015), and distant lymph node metastasis (*p* < 0.000) remained independently associated with BM (Table 3).

**TABLE 3:** Univariate and multivariable logistic regression analysis of variables.

Variables	Category	Univariate analysis		Multivariable analysis	
		Odds ratio (95% CI)	*p* value	Odds ratio (95% CI)	*p* value
Age	\	0.980 (0.973–0.987)	< 0.000*	0.981 (0.975–0.988)	< 0.000*
Gender	Male	Ref	Ref	Ref	Ref
	Female	0.97 7 (0.862–1.107)	0.715	\	\
Race	White	0.898 (0.751–1.075)	0.242	\	\
	Black	1.184 (0.954–1.470)	0.125	\	\
	Other	0.981 (0.733–1.312)	0.896	\	\
Marital status	Married (including common law)	1.033 (0.911–1.171)	0.614	1.016 (0.845–1.223)	0.865
	Divorced	1.019 (0.858–1.211)	0.830	1	Ref
	Separated	0.964 (0.577–1.609)	0.887	0.910 (0.531–1.560)	0.733
	Single (never married)	1.150 (0.978–1.352)	0.090	1.041 (0.836–1.296)	0.719
	Unmarried or domestic partner	1.139 (0.524–2.479)	0.742	0.992 (0.443–2.219)	0.984
	Widowed	0.790 (0.661–0.943)	0.009*	0.923 (0.729–1.169)	0.504
T stage	T1	0.655 (0.551–0.779)	< 0.000*	1	Ref
	T2	1.009 (0.861–1.183)	0.910	1.446 (1.157–1.808)	0.001*
	T3	0.959 (0.818–1.125)	0.611	1.352 (1.046–1.749)	0.021*
	T4	1.308 (1.153–1.484)	< 0.000*	1.710 (1.303–2.246)	< 0.001*
N stage	N0	0.826 (0.696–0.979)	0.028*	1	Ref
	N1	0.851 (0.680–1.066)	0.160	0.964 (0.736–1.261)	0.786
	N2	1.018 (0.898–1.154)	0.785	1.008 (0.838–1.213)	0.933
	N3	1.205 (1.046–1.387)	0.010*	0.961 (0.779–1.185)	0.707
Tumor size	\	1.002 (1.000–1.004)	0.048*	0.997 (0.993–1.000)	0.046*
Separate tumor nodules ipsilateral lung	None	0.793 (0.690–0.912)	0.001	1.146 (0.851–1.543)	0.370
	Different lobe	1.106 (0.847–1.445)	0.460	1	Ref
	Same lobe	1.131 (0.919–1.393)	0.245	1.231 (0.865–1.752)	0.248
	Same and different lobes	1.267 (0.931–1.723)	0.133	1.027 (0.684–1.543)	0.897
	Unknown if same or different lobe	1.339 (1.063–1.687)	0.013*	1.272 (0.895–1.808)	0.179
Mets at DX‐bone	No	Ref	Ref	Ref	Ref
	Yes	1.495 (1.302–1.715)	< 0.000*	1.283 (1.103–1.492)	0.001*
Mets at DX‐liver	No	Ref	Ref	Ref	Ref
	Yes	1.259 (1.102–1.439)	0.001*	1.011 (0.873–1.170)	0.886
Mets at DX‐lung	No	Ref	Ref	Ref	Ref
	Yes	1.583 (1.322–1.895)	< 0.000*	1.286 (1.051–1.575)	0.015*
Mets at DX‐Distant LN	No	Ref	Ref	Ref	Ref
	Yes	2.055 (1.748–2.415)	< 0.000*	1.773 (1.492–2.107)	< 0.000*

*Note:* Other race: American Indian/AK Native, Asian/Pacific Islander.

Abbreviations: At DX, at the time of diagnosis; Mets, metastasis; LN, lymph nodes.

* *p* < 0.05.

### 3.3 Correlation Analysis

**FIGURE 2:**
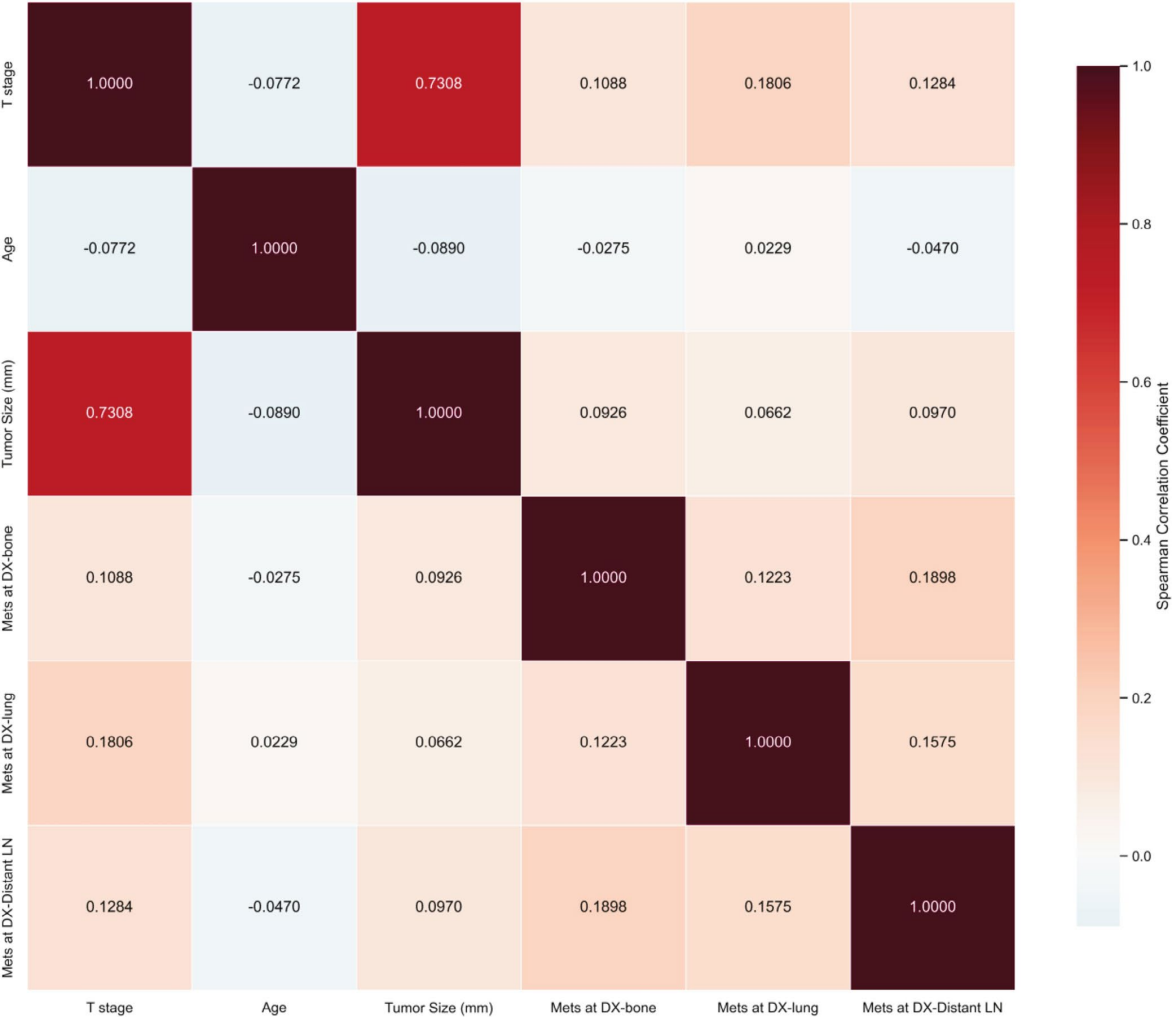
A heatmap representation of the Spearman correlation matrix of the variables. Relevant correlations are color‐coded based on the strength of the correlation. At DX, at the time of diagnosis; LN, lymph nodes; Mets, metastasis; Mm, millimeter.

The Spearman correlation coefficients among the predictors are presented in Figure 2. A strong correlation was observed between T stage and tumor size (ρ = 0.7308). All other correlations among the predictors were weak (|ρ| < 0.5).

### 3.4 Model Performance

**FIGURE 3:**
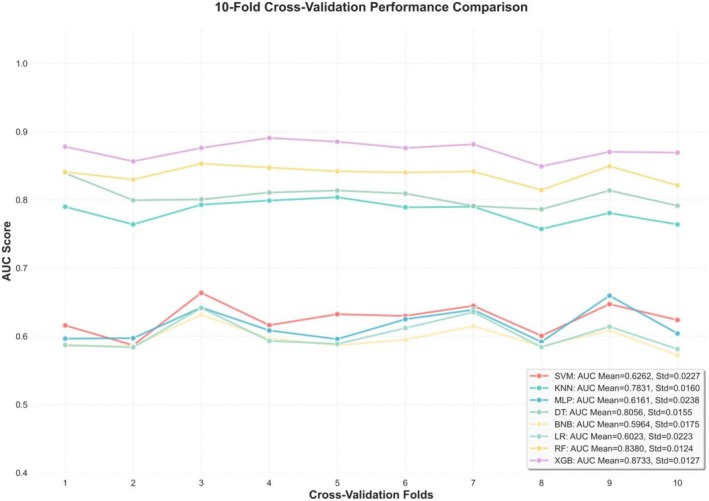
AUC values of 10‐fold cross‐validation. The AUC was used as an indicator of performance; the XGB model achieved the best predictive performance. AUC, area under the curve; BNB, Bernoulli Naïve Bayes; DT, decision tree; KNN, K‐nearest neighbor; LR, logistic regression; MLP, multilayer perceptron; RF, random forest; Std, standard deviation; SVM, support vector machine; XGB, extreme gradient boosting machine.

**FIGURE 4:**
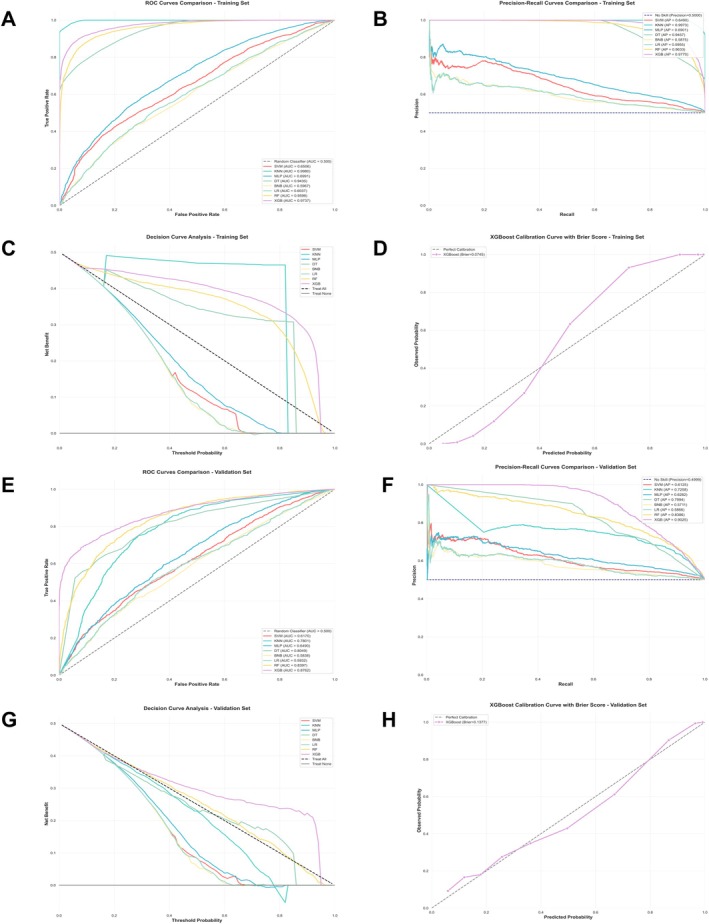
ROC, PR, calibration, and decision‐curve plots. (A) ROC curves of different machine learning models in the training set. (B) PR curves of different machine learning models in the training set. (C) DCA curves of different machine learning models in the training set. (D) Calibration curves of different machine learning models in the training set. (E) ROC curves of different machine learning models in the validation set. (F) PR curves of different machine learning models in the validation set. (G) DCA curves of different machine learning models in the validation set. (H) Calibration curves of different machine learning models in the validation set. AP, AUPRC, area under the precision‐recall curve; BNB, Bernoulli Naïve Bayes; DCA, decision curve analysis; DT, decision tree; KNN, K‐nearest neighbor; LR, logistic regression; MLP, multilayer perceptron; PR, precision‐recall; RF, random forest; ROC, receiver operating characteristic; SVM, support vector machine; XGB, extreme gradient boosting machine.

**FIGURE 5:**
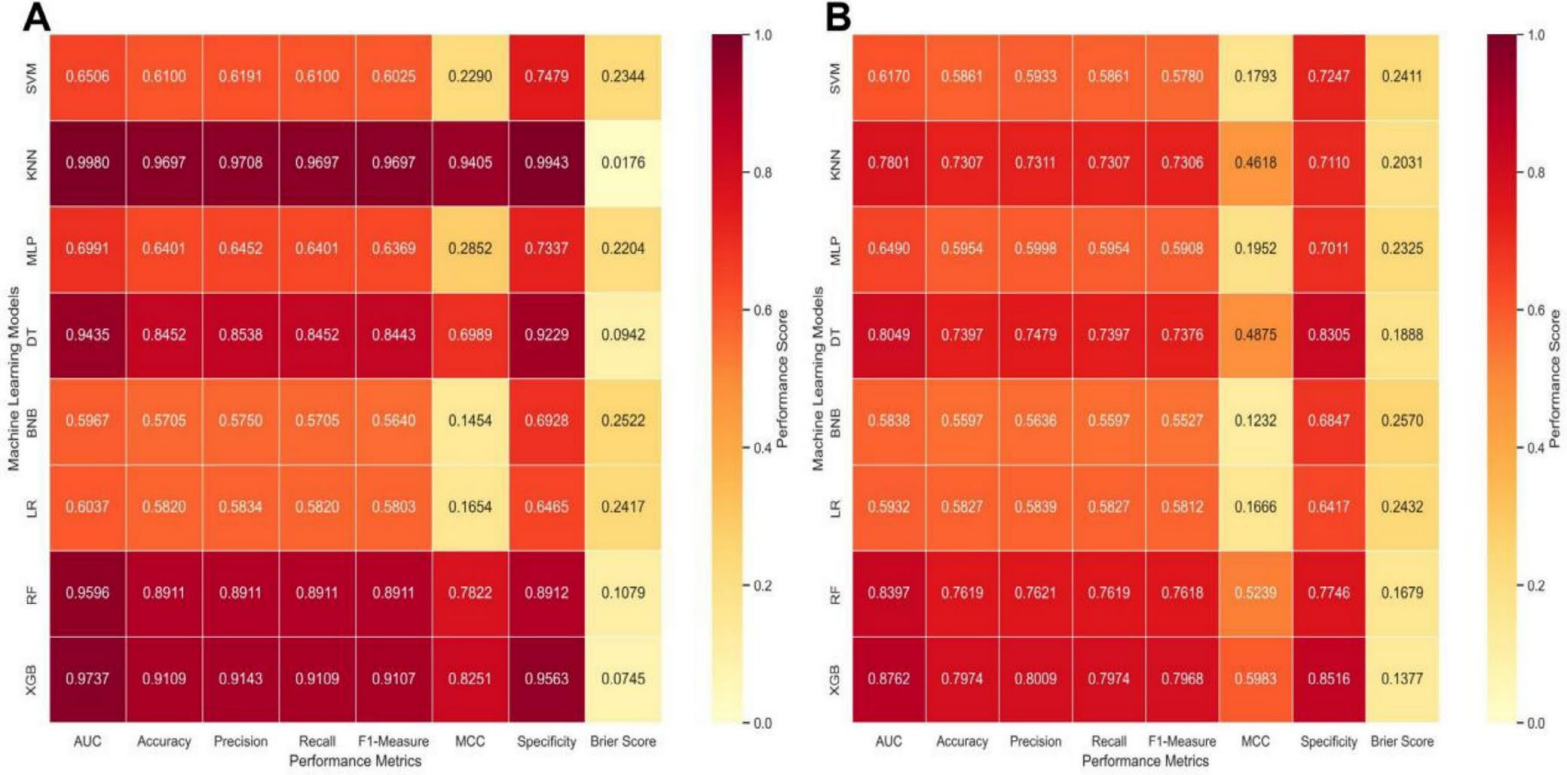
(A) Prediction performance of different models in the training set. (B) Prediction performance of different models in the validation set. AUC, area under the curve; BNB, Bernoulli Naïve Bayes; DT, decision tree; F1, F1 score; KNN, K‐nearest neighbor; LR, logistic regression; MCC, Matthews correlation coefficient; MLP, multilayer perceptron; RF, random forest; SVM support vector machine; XGB, extreme gradient boosting machine.

The 10‐fold cross‐validation results for all ML models are shown in Figure 3. Among the eight algorithms evaluated, XGB achieved the highest mean cross‐validated AUC (0.8733, SD = 0.0127), outperforming RF (0.8380, SD = 0.0124), DT (0.8056, SD = 0.0155), KNN (0.7831, SD = 0.0160), SVM (0.6262, SD = 0.0227), MLP (0.6161, SD = 0.0238), LR (0.6023, SD = 0.0223), and BNB (0.5964, SD = 0.0175). In the training set, XGB also demonstrated the excellent AUPRC (0.9770) (Table 4, Figure 4B). Performance metrics in the validation cohort are provided in Table 4, with corresponding ROC, PR, calibration, and decision‐curve plots shown in Figures 4 and 5. In the validation set, XGB achieved an AUC of 0.8762, accuracy of 0.7974, precision of 0.8009, recall of 0.7974, specificity of 0.8516, MCC of 0.5983, F1‐score of 0.7968, the highest AUPRC of 0.9025 and lowest Brier score of 0.1377 among all models. Based on its superior discrimination, calibration, and clinical utility, the XGB algorithm was selected as the final prediction model.

**TABLE 4:** Performance comparison of eight ML models in training and validation set.

Set	Model	AUC	Accuracy	Precision	Recall	F1‐score	MCC	Brier score	AUPRC
Training	XGB	0.9737	0.9109	0.9143	0.9109	0.9107	0.8251	0.0745	0.9770
	RF	0.9596	0.8911	0.8911	0.8911	0.8911	0.7822	0.1079	0.9633
	DT	0.9435	0.8452	0.8538	0.8452	0.8443	0.6989	0.0942	0.9457
	KNN	0.9980	0.9697	0.9708	0.9697	0.9697	0.9405	0.0176	0.9973
	MLP	0.6991	0.6401	0.6452	0.6401	0.6369	0.2852	0.2204	0.6901
	SVM	0.6506	0.6100	0.6191	0.6100	0.6025	0.2290	0.2344	0.6490
	LR	0.6037	0.5820	0.5834	0.5820	0.5803	0.1654	0.2417	0.5955
	BNB	0.5967	0.5705	0.5750	0.5705	0.5640	0.1454	0.2522	0.5875
Validation	XGB	0.8762	0.7974	0.8009	0.7974	0.7968	0.5983	0.1377	0.9025
	RF	0.8397	0.7619	0.7621	0.7619	0.7618	0.5239	0.1679	0.8386
	DT	0.8049	0.7397	0.7479	0.7397	0.7376	0.4875	0.1888	0.7994
	KNN	0.7801	0.7307	0.7311	0.7307	0.7306	0.4618	0.2031	0.7258
	MLP	0.6490	0.5954	0.5998	0.5954	0.5908	0.1952	0.2325	0.6282
	SVM	0.6170	0.5861	0.5933	0.5861	0.578	0.1793	0.2411	0.6125
	LR	0.5932	0.5827	0.5839	0.5827	0.5812	0.1666	0.2432	0.5866
	BNB	0.5838	0.5597	0.5636	0.5597	0.5527	0.1232	0.2570	0.5711

Abbreviations: AUC, area under the curve; AUPRC, area under the precision‐recall curve; BNB, Bernoulli Naïve Bayes; DT, decision tree; KNN, K‐nearest neighbor; LR, logistic regression; MCC, Matthews correlation coefficient; ML, machine learning; MLP, multilayer perceptron; RF, random forest; SVM, support vector machine; XGB, extreme gradient boosting machine.

The calibration performance in the validation set was satisfactory. The calibration intercept was 0.0211, and the calibration slope was 0.9871, which were close to the ideal values of 0 and 1, indicating no substantial systematic overestimation or underestimation of the predicted risk. The Brier score was 0.1377, reflecting good overall predictive accuracy and low prediction error. Although the Hosmer–Lemeshow test was statistically significant (*χ* ^2^ = 55.57, *p* < 0.001), this result was likely influenced by the large sample size, which may have increased the sensitivity to minor and clinically unimportant deviations from perfect calibration. Overall, the XGBoost model demonstrated good calibration performance in the validation cohort.

### 3.5 External Validation of the Model

**FIGURE 6:**
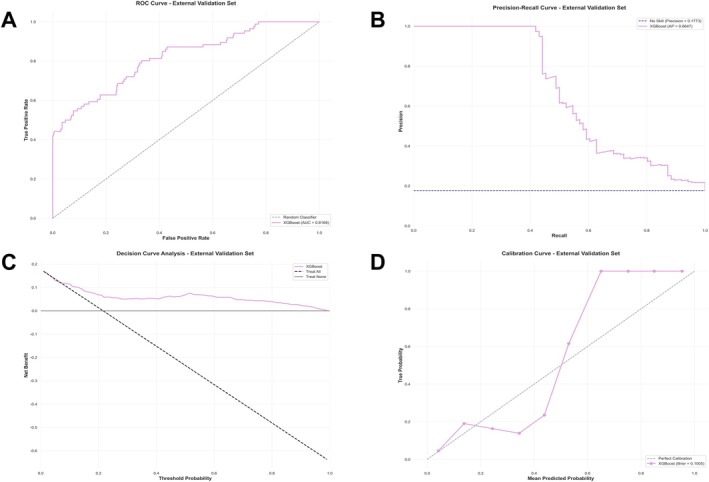
ROC, PR, calibration, and decision‐curve plots of XGB model in the external validation set. (A) ROC curves. (B) PR curves. (C) DCA curves. (D) Calibration curves. AP, AUPRC: area under the precision‐recall curve; DCA, decision curve analysis; PR, precision‐recall; ROC, receiver operating characteristic; XGB, extreme gradient boosting machine.

In the external validation cohort consisting of 485 cases (Table 5), the XGB model achieved an AUC of 0.8169, accuracy of 0.8907, precision of 0.8837, recall of 0.4419, MCC of 0.5769, F1‐score of 0.5891, and AUPR of 0.6647 (Figure 6A,B, Table S3). The calibration curve revealed that the XGB model had a lower Brier score (0.1005) (Figure 6D). DCA and clinical impact curves confirmed that the XGB model has potential clinical utility and reliable predictive performance (Figure 6C,D).

**TABLE 5:** Clinical characteristics of external validation set.

Variables	All	BM	NBM	*p* value
	*N* = 485	*N* = 86	*N* = 399	
Age (mean (SD))	64.66 (8.59)	64.09 (8.02)	64.79 (8.71)	0.4751
Gender, *n* (%)				0.021*
Male	111 (22.89)	11 (12.79)	100 (25.06)	
Female	374 (77.11)	75 (87.21)	299 (74.94)	
Race, *n* (%)				1
Other	485 (100)	86 (100)	399 (100)	
Marital status, *n* (%)				0.218
Divorced	1 (0.21)	0	1 (0.25)	
Married (including common law)	455 (93.81)	80 (93.02)	375 (93.98)	
Separated	1 (0.21)	1 (1.16)	0	
Single (never married)	5 (1.03)	2 (2.33)	3 (0.75)	
Widowed	22 (4.54)	3 (3.49)	19 (4.76)	
Unknown	1 (0.21)	0	1 (0.25)	
T stage, *n* (%)				0.115
T1	60 (12.37)	7 (8.14)	53 (13.28)	
T2	68 (14.02)	8 (9.30)	60 (15.04)	
T3	72 (14.85)	18 (20.93)	54 (13.53)	
T4	285 (58.76)	53 (60.63)	232 (58.15)	
N stage, *n* (%)				0.24
N0	84 (17.32)	13 (15.12)	71 (17.79)	
N1	25 (5.15)	5 (5.81)	20 (5.01)	
N2	211 (43.51)	31 (36.05)	180 (45.11)	
N3	165 (34.02)	37 (43.02)	128 (32.08)	
Tumor size (mean (SD)) (mm)	59.55 (29.50)	64.33 (31.79)	58.52 (28.92)	0.1215
Separate tumor nodules ipsilateral lung, *n* (%)				0.164
None	360 (74.23)	54 (62.79)	306 (76.69)	
Same lobe	54 (11.13)	14 (16.28)	40 (10.03)	
Different lobe	20 (4.12)	5 (5.81)	15 (3.76)	
Same and different lobes	22 (4.54)	6 (6.98)	16 (4.01)	
Unknown if same or different lobe	22 (4.54)	6 (6.98)	16 (4.01)	
Not documented	7 (1.44)	1 (1.16)	6 (1.5)	
Mets at DX‐bone, *n* (%)				0.204
No	393 (81.03)	65 (75.58)	328 (82.21)	
Yes	92 (18.97)	21 (24.42)	71 (17.79)	
Mets at DX‐liver, *n* (%)				0.293
No	384 (79.18)	64 (74.42)	320 (80.2)	
Yes	101 (20.82)	22 (25.58)	79 (19.8)	
Mets at DX‐lung, *n* (%)				< 0.002*
No	424 (87.42)	66 (76.74)	358 (89.72)	
Yes	61 (12.58)	20 (23.26)	41 (10.28)	
Mets at DX‐Distant LN, *n* (%)				< 0.001*
No	414 (85.36)	63 (73.26)	351 (87.97)	
Yes	71 (14.64)	23 (26.74)	48 (12.03)	

*Note:* Other race: American Indian/AK Native, Asian/Pacific Islander.

Abbreviations: At DX: at the time of diagnosis; BM: brain metastasis; LN: lymph nodes; Mets: metastasis; mm: millimeter; NBM: no brain metastasis.

* *p* < 0.05.

DCA was performed in the external validation cohort, which had a brain metastasis prevalence of 17.7%. Within the clinically relevant threshold probability range of 0.10–0.50, the XGB model demonstrated a consistent positive net benefit and outperformed the “treat‐all” strategy. Notably, when the threshold probability exceeded 0.20, the net benefit of the “treat‐all” strategy became negative, whereas the XGB model maintained a stable, positive net benefit. These findings suggest that the model may provide potential clinical utility for exploratory risk stratification in an external validation cohort.

### 3.6 Feature Importance and SHAP Analysis

**FIGURE 7:**
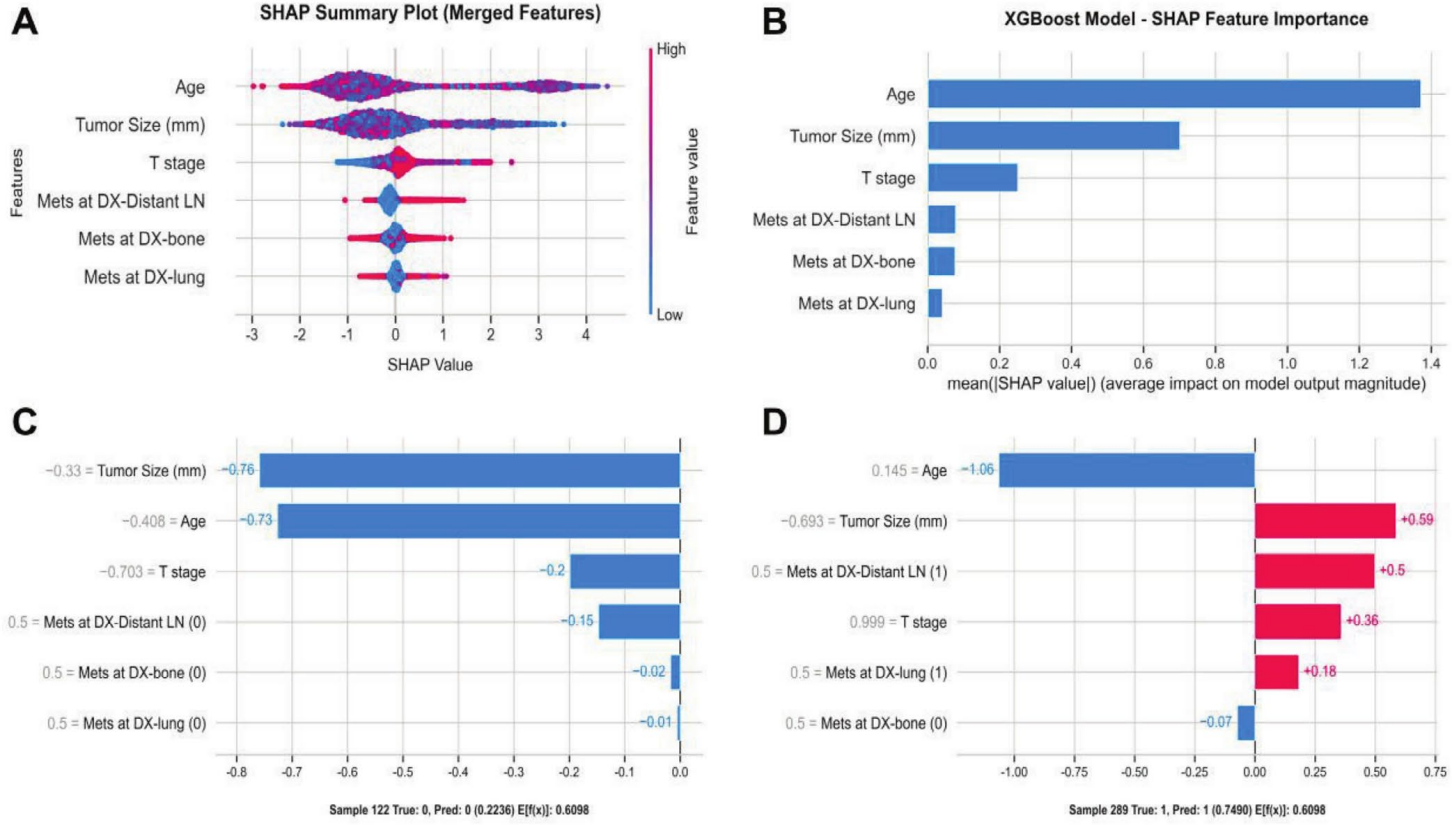
SHAP summary plot and SHAP model explanation of two typical predictions. (A) showed the SHAP summary plot. The features are ranked according to the sum of the SHAP values for all patients, and the SHAP values are used to show the distribution of the effect of each feature on the XGB model outputs. Each dot represents a case in the dataset. The color of a dot indicates the value of the feature, with blue indicating the lowest range and red the highest range. The horizontal axis shows the corresponding SHAP value of the feature. A positive SHAP value contributes to the prediction of rupture and vice versa. The baseline predicted value of XGB model is 0.6098. (B) showed the SHAP feature importance of the XGB model. (C) showed the low‐risk SHAP interpretation model for patients with no brain metastasis of SCLC. This sample had a predicted value of 0.2236, with T stage, lung metastasis, tumor size, distant lymph node metastasis, bone metastasis, and age contributing to the decrease in the predicted value. (D) showed the SHAP interpretation model of high‐risk in patients with brain metastasis of SCLC. This sample had a predicted value of 0.7490, with age and bone metastasis reduced the predicted value, while tumor size, distant lymph node metastasis, T stage, and lung metastasis increased it. 0, no brain metastasis; 1, brain metastasis; At DX, at the time of diagnosis; *E*[*f*(*x*)], the average predicted value of the model for all samples; LN, lymph nodes; Mets, metastasis; mm, millimeter; Pred, predicted value; SCLC, small‐cell lung cancer; SHAP, Shapley additive interpretation; XGBoost, XGB, extreme gradient boosting machine.

The global feature importance derived from the XGB model is displayed in Figure 7A,B, with the largest importance values observed for age, tumor size, T stage, distant lymph node metastasis, bone metastasis, and lung metastasis. SHAP summary and dependence plots are provided in Figure S1. Two representative individual‐level SHAP waterfall plots are shown in Figure 7C,D, demonstrating the calculated contribution of each predictor to the final predicted BM probability for low‐risk and high‐risk cases. These analyses illustrate predictor contributions at the global and individual levels but are not used for causal or mechanistic interpretation.

### 3.7 Web‐Based Risk Calculator

**FIGURE 8:**
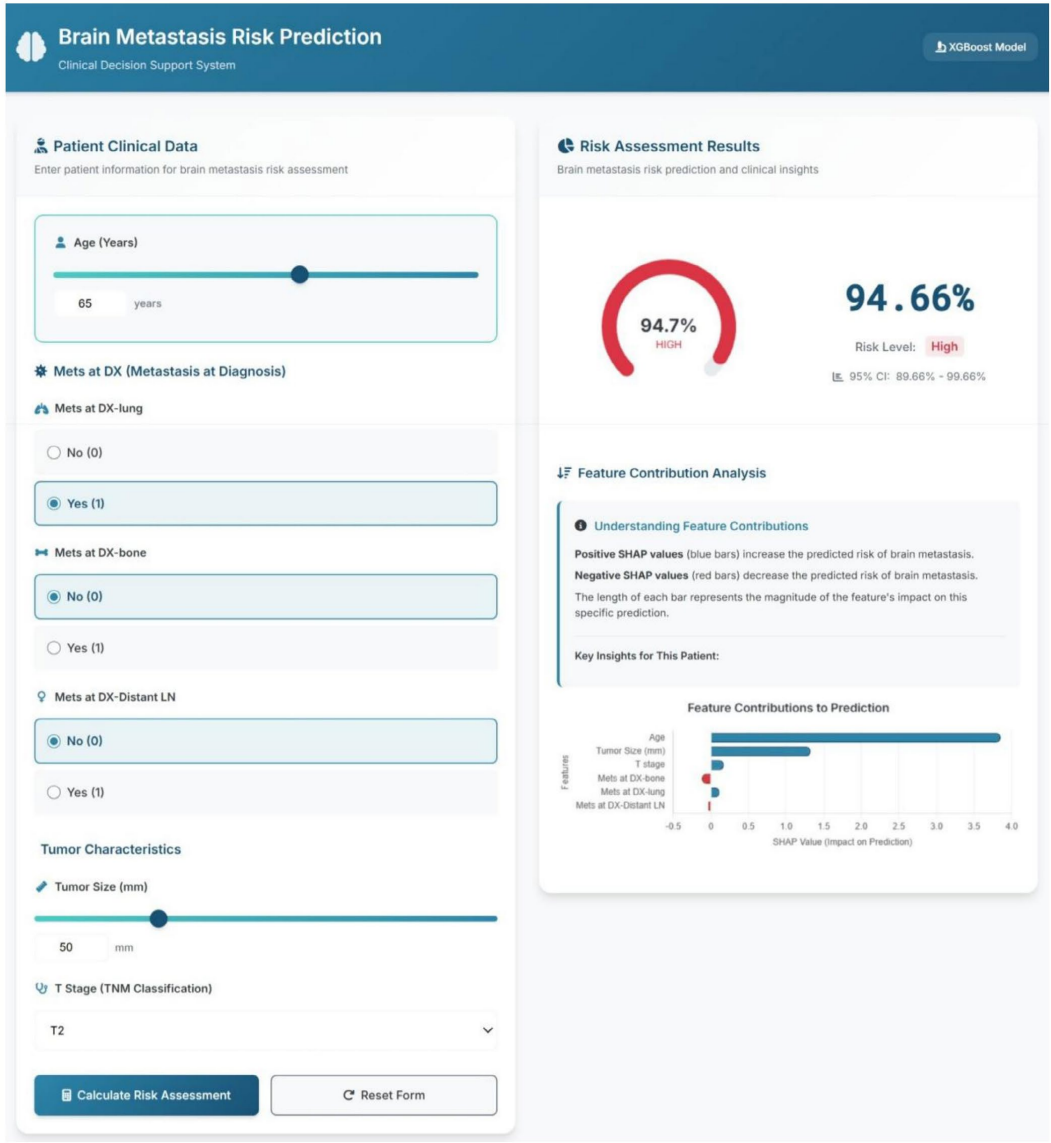
Web calculator for predicting brain metastasis of SCLC. URL was http://101.37.164.15:7778/. At DX, at the time of diagnosis; LN, lymph nodes; Mets, metastasis; mm, millimeter; SCLC, small cell lung cancer; SHAP, Shapley additive interpretation; XGBoost, XGB, extreme gradient boosting machine.

To facilitate the accessibility and exploratory application of the model, a web‐based calculator (http://101.37.164.15:7778/) was developed using the final XGB model. Users can input routinely available clinical variables to obtain individualized risk estimates for brain metastasis at diagnosis. However, this tool is intended for research and exploratory risk stratification purposes only and requires further prospective multicenter validation before routine clinical implementation. Screenshots of the interface are shown in Figure 8.

## 4 Discussion

BM is associated with substantial morbidity and mortality in SCLC, underscoring the need for the early identification of patients at elevated risk. Although brain MRI is recommended at diagnosis and during symptomatic follow‐up, current guidelines do not advocate routine surveillance MRI in asymptomatic patients [25]. Therefore, a reliable prediction model may help stratify patients who warrant intensified surveillance or preventive interventions, enabling earlier detection of occult BM and potentially reducing treatment delays and the healthcare burden.

In this study, we developed and validated multiple ML models to predict BM risk in the overall SCLC population and identified XGB as the best performing algorithm. XGB demonstrated robust discrimination and calibration, which is consistent with prior reports of its superior performance in handling non‐linear interactions, high‐dimensional data, and noise [26, 27, 28, 29]. Our findings extend the previous work of Munai et al. [7], who developed ML models exclusively for extensive‐stage SCLC (ES‐SCLC). By incorporating both limited‐stage and extensive‐stage diseases, the present study provides a more comprehensive tool that reflects real‐world clinical populations.

A major challenge limiting the clinical uptake of ML models is their “black‐box” nature [30]. To address this, we applied SHAP, which enhances transparency by quantifying each variable's contribution to the final prediction at both the global and individual levels [31, 32]. SHAP visualizations demonstrated the relative influence of age, tumor burden–related factors, and metastatic patterns on BM risk, thereby improving interpretability and supporting the clinical acceptance of the model.

Logistic regression analysis identified several BM‐associated factors, including age, T stage, tumor size, bone metastasis, lung metastasis, and distant lymph node metastasis. These associations are largely consistent with those in previous studies [33, 34, 35, 36, 37, 38, 39, 40, 41]. Younger age has been repeatedly associated with higher BM risk, possibly reflecting biological differences in metastatic tropism or competing mortality in older patients [33, 34, 35, 36]. Tumor size and advanced T stage have also been strongly linked to BM development; larger primary tumors may shed more circulating tumor cells capable of colonizing the central nervous system [37, 38, 39]. Distant lymph node involvement, particularly N3 disease, further increases the likelihood of distant spread, including BM, a phenomenon supported by mechanistic evidence implicating the CXCL12/CXCR4 axis in metastasis [42, 43, 44, 45].

The clinical management of BM in SCLC remains challenging, particularly regarding the role of PCI. Historically, PCI has been considered a standard strategy for selected patients with limited‐stage SCLC who respond to chemoradiotherapy [46]. However, more recent MRI‐based surveillance studies have questioned its overall survival benefit and highlighted the potential for neurocognitive toxicity [47, 48, 49]. In this context, there is increasing interest in identifying patients with a higher likelihood of BM using routinely available clinical variables [46, 50]. Although brain MRI remains the standard approach for staging evaluation in SCLC, the present model may provide supplementary risk stratification information at the initial diagnosis and support individualized imaging assessment and exploratory clinical decision‐making. However, prospective validation is still required before the model can be incorporated into routine clinical management or used to guide PCI‐related decision‐making.

This study has several strengths. First, it uses a large, contemporary SEER cohort with broad population coverage, enhancing generalizability. Second, multiple ML algorithms were compared using rigorous cross validation. Third, the SHAP methodology provides interpretable insights that are essential for clinical implementation.

Multiple artificial intelligence‐based studies have addressed clinical challenges related to lung cancer brain metastasis, but they vary greatly in research objectives and technical schemes. Kuzan et al. [51] developed decision tree models based on high‐dimensional radiomics features extracted from contrast‐enhanced brain MRI, which accurately predicted common gene mutations in NSCLC patients with BM. This method avoids invasive tissue biopsy and serves targeted therapy decision‐making for NSCLC. Nevertheless, the radiomics model is limited by high requirements for imaging standardization, and it cannot be used for early risk screening before confirming metastatic lesions. Our study targeted SCLC and developed an interpretable XGB model with routine clinical data to predict synchronous BM at initial diagnosis. Equipped with a web‐based calculator, our tool enables convenient early risk stratification for general clinical use. Collectively, the radiomics‐based molecular prediction and our clinical‐data‐based metastasis risk prediction represent two complementary directions. Combining clinical characteristics and imaging omics data will help establish a more comprehensive evaluation system for lung cancer brain metastasis.

This study has several limitations. First, because the SEER database records metastatic status at diagnosis, the present model primarily identifies patients with synchronous brain metastasis rather than predicting future BM development during follow‐up. Second, the retrospective nature of the study may introduce potential selection and information bias. Third, SEER lacks detailed treatment‐related information, including chemotherapy regimens, immunotherapy, radiotherapy parameters, prophylactic cranial irradiation use, MRI surveillance frequency, treatment response, and performance status. In addition, smoking history, molecular biomarkers, laboratory parameters, and imaging features were unavailable. The absence of these variables may influence both the occurrence and detection of brain metastasis and limit further evaluation of treatment‐related effects and biological predictors. Fourth, the use of complete‐case analysis by excluding patients with missing data may have introduced potential selection bias. Although statistically significant differences in age and marital status were observed between the training and validation cohorts, the absolute differences were relatively small, and stable model performance across internal and external validation analyses suggests limited impact on overall predictive performance. Future studies integrating radiomics, genomics, proteomics, and other multi‐omics data with clinical variables may further improve the biological interpretability and predictive performance of the model.

## 5 Conclusions

Using a large population‐based cohort, we developed and validated multiple ML models for identifying brain metastasis at diagnosis in patients with SCLC, with XGB demonstrating the best predictive performance. The final model, supported by SHAP‐based interpretability, enables individualized risk estimation using routinely available clinical variables. This tool may provide supplementary risk stratification information for patients with a higher likelihood of brain metastasis at diagnosis and support exploratory clinical assessments. However, further prospective multicenter validation is required before routine clinical implementation. Additional external validation and prospective evaluation are also needed to assess the model's applicability across diverse clinical settings and explore its integration with emerging imaging and molecular biomarkers.

## Author Contributions

**Zi‐Kun Huang:** visualization, software, methodology, validation. **Yong Zhai:** data curation, validation, visualization. **Xian‐Ling Zhao:** data curation, validation, visualization. **Yu‐Long He:** conceptualization, funding acquisition, project administration, data curation, formal analysis, methodology, resources, validation, visualization, writing – review and editing. **Qin‐Ling Jiang:** data curation, formal analysis, methodology, resources, validation, visualization, writing – original draft.

## Funding

Self‐raised Funds Research Project of the Guangxi Zhuang Autonomous Region Health Commission (Z‐C20250176); Discipline Group Construction Fund of Nanxishan Hospital of Guangxi Zhuang Autonomous Region; Guangxi Medical and Health Appropriate Technology Development and Application Project (S2025012).

## Ethics Statement

The Institutional Review Board of Nanxishan Hospital of the Guangxi Zhuang Autonomous Region approved this study (approval number NXSYY‐2025‐053(Y), approval date March 17, 2025).

## Conflicts of Interest

The authors declare no conflicts of interest.

## Supporting information


**Figure S1:** The importance of variables in each prediction model. (A) Feature importance of SVM. (B) Feature importance of BNB. (C) Feature importance of KNN. (D) Feature importance of LR. (E) Feature importance of MLP. (F) Feature importance of RF. (G) Feature importance of DT. (H) Feature importance of XGB. At DX, at the time of diagnosis; BNB, Bernoulli Naïve Bayes; DT, decision tree; KNN, K‐nearest neighbor; LN, lymph nodes; LR, logistic regression; Mets, metastasis; MLP, multilayer perceptron; RF, random forest; SVM, support vector machine; XGB, extreme gradient boosting machine.

**Table S1:** Normalization standards.

**Table S2:** Property values of clinical features in models.

**Table S3:** Performance of XGB model in the external validation set.

## Data Availability

All data used in this work can be acquired from the Surveillance, Epidemiology, and End Results Program (SEER; https://seer.cancer.gov/SEER). The source code for this paper's experiments and analyses is publicly available (https://github.com/Huangzikun/BrainMets).
