# Supplementary material for: Machine Learning‐Based Prediction of Brain Metastasis at Initial Diagnosis in Small‐Cell Lung Cancer: Model Development and SHAP Interpretation Study
Source: Cancer Rep (Hoboken). 2026 Jul 16;9(7):e70625. doi: 10.1002/cnr2.70625 (PMC13374636; doi:10.1002/cnr2.70625)
Supplement: Supplementary file 1 — Figure S1: The importance of variables in each prediction model. (A) Feature importance of SVM. (B) Feature importance of BNB. (C) Feature importance of KNN. (D) Feature importance of LR. (E) Feature importance of MLP. (F) Feature importance of RF. (G) Feature importance of DT. (H) Feature importance of XGB. At DX, at the time of diagnosis; BNB, Bernoulli Naïve Bayes; DT, decision tree; KNN, K‐nearest neighbor; LN, lymph nodes; LR, logistic regression; Mets, metastasis; MLP, multilayer perceptron; RF, random forest; SVM, support vector machine; XGB, extreme gradient boosting machine. [file CNR2-9-e70625-s004.docx]

**
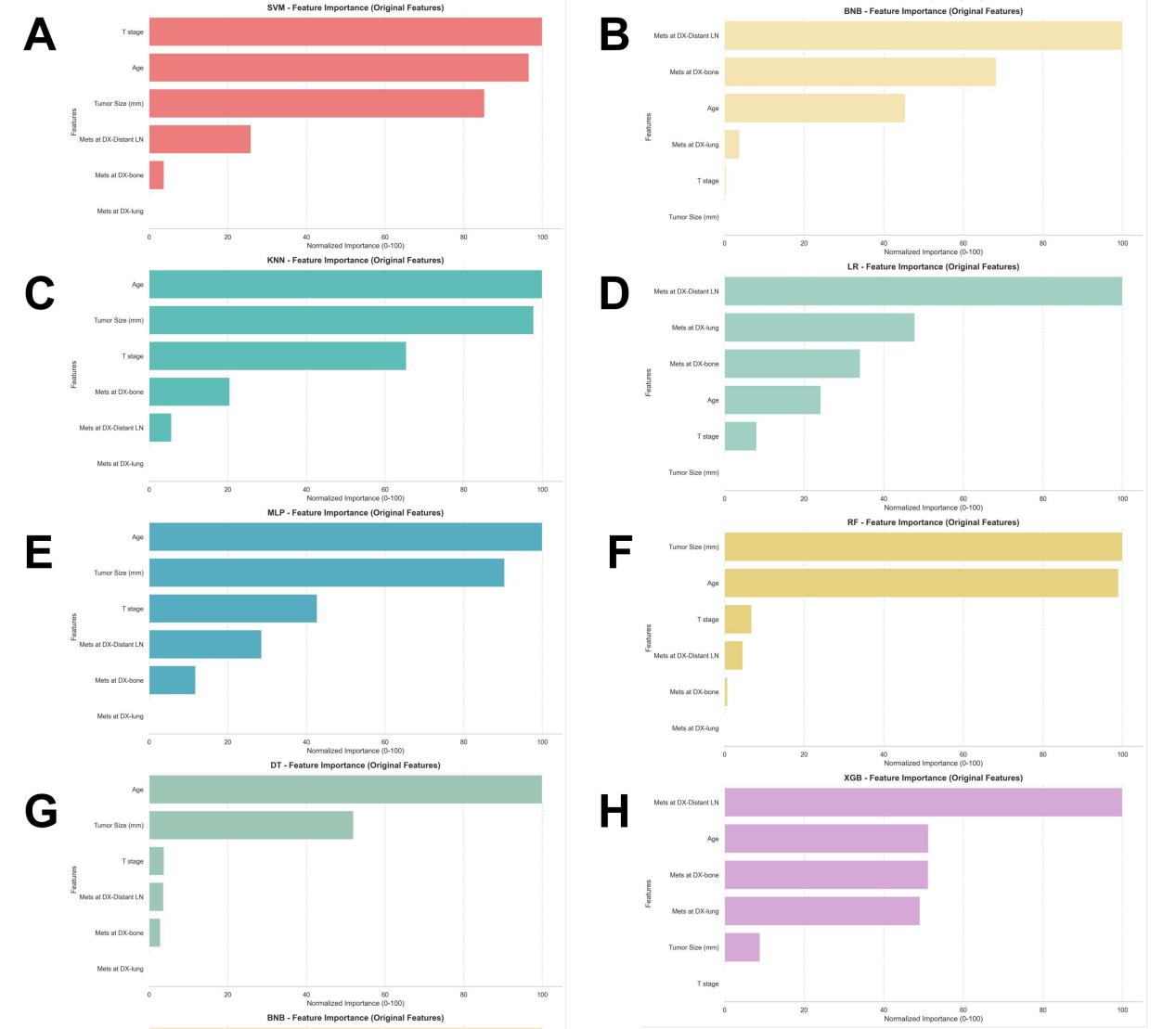
**

**Supplement Figure S1.** The importance of variables in each prediction model. **(A)** Feature importance of SVM. **(B)** Feature importance of BNB. **(C)** Feature importance of KNN. **(D)** Feature importance of LR. **(E)** Feature importance of MLP. **(F)** Feature importance of RF. **(G)** Feature importance of DT. **(H)** Feature importance of XGB.**Abbreviation:** LR, logistic regression; XGB, extreme gradient boosting machine; BNB, Bernoulli Naïve Bayes; RF, random forest; DT, decision tree; MLP, multilayer perceptron; KNN, K-nearest neighbor; SVM, support vector machine; Mets, metastasis; At DX, at the time of diagnosis; LN, lymph nodes.
