# Supplementary material for: Machine Learning‐Based Prediction of Brain Metastasis at Initial Diagnosis in Small‐Cell Lung Cancer: Model Development and SHAP Interpretation Study
Source: Cancer Rep (Hoboken). 2026 Jul 16;9(7):e70625. doi: 10.1002/cnr2.70625 (PMC13374636; doi:10.1002/cnr2.70625)
Supplement: Supplementary file 2 — Table S1: Normalization standards. [file CNR2-9-e70625-s003.docx]

**Supplement Table S1.** Normalization standards.

| **Clinical data** | **SEER normalization standard** |
| --- | --- |
| **Basic demographic data** |  |
| Age | Age at diagnosis |
| Gender | Gender |
| Race | Race |
| Marital status | Marital status at diagnosis |
| **Tumor information** |  |
| TNM stage | According to AJCC 8th edition |
| Tumor size(mm) | the largest diameter of the primary tumor |
| Show one or more satellite nodules in the same pulmonary lobe | Separate tumor nodules ipsilateral lung |
| Visceral and parietal pleural invasion | Visceral and parietal pleural invasion |
| Combined with bone metastasis | Mets at DX-bone |
| Combined with liver metastasis | Mets at DX-liver |
| Combined with lung metastasis | Mets at DX-lung |
| Combined with distant lymph nodes metastasis | Mets at DX-Distant LN |
| Brain metastasis | Mets at DX-brain |

***Abbreviation:*** SEER: the population-based Surveillance, Epidemiology, and End Results; AJCC, American Joint Committee on Cancer; mm, millimeter; Mets, metastasis; At DX, at the time of diagnosis; LN, lymph nodes.
