# Supplementary material for: Machine Learning‐Based Prediction of Brain Metastasis at Initial Diagnosis in Small‐Cell Lung Cancer: Model Development and SHAP Interpretation Study
Source: Cancer Rep (Hoboken). 2026 Jul 16;9(7):e70625. doi: 10.1002/cnr2.70625 (PMC13374636; doi:10.1002/cnr2.70625)
Supplement: Supplementary file 3 — Table S2: Property values of clinical features in models. [file CNR2-9-e70625-s002.docx]

**Supplement Table S2.** Property values of clinical features in models.

| **Variables** | **Property Values** |
| --- | --- |
| Gender |  |
| Male | 0 |
| Female | 1 |
| Race |  |
| White | 0 |
| Black | 1 |
| Other (American Indian/AK Native, Asian/Pacific Islander) | 2 |
| Marital status |  |
| Divorced | 0 |
| Married (including common law) | 1 |
| Separated | 2 |
| Single (never married) | 3 |
| Unmarried or domestic partner | 4 |
| Widowed | 5 |
| T stage |  |
| T1 | 0 |
| T2 | 1 |
| T3 | 2 |
| T4 | 3 |
| N stage |  |
| N0 | 0 |
| N1 | 1 |
| N2 | 2 |
| N3 | 3 |
| Separate tumor nodules ipsilateral lung |  |
| None; No intrapulmonary mets; Foci in situ/minimally  invasive adenocarcinoma | 0 |
| Separate nodules of same hist type in ipsilateral lung, same  lobe | 1 |
| Separate nodules of same hist type in ipsilateral lung,  different lobe | 2 |
| Separate nodules of same hist type in ipsilateral lung, same  AND different lobes | 3 |
| Separate tumor nodules, ipsilateral lung, unknown if same  or different lobe | 4 |
| Visceral and Parietal Pleural Invasion |  |
| PL0; No evidence; Tumor does not completely traverse the elastic layer of pleura | 0 |
| PL1 or PL2; Invasion of visceral pleura present, NOS | 1 |
| PL3; Tumor invades into or through the parietal pleura OR chest wall | 2 |
| Tumor extends to pleura, NOS; not stated if visceral or  parietal | 3 |
| Not documented; No resection of primary; Not assessed or unknown if assessed | 4 |
| Mets at DX-bone |  |
| No | 0 |
| Yes | 1 |
| Mets at DX-liver |  |
| No | 0 |
| Yes | 1 |
| Mets at DX-lung |  |
| No | 0 |
| Yes | 1 |
| Mets at DX-Distant LN |  |
| No | 0 |
| Yes | 1 |
| Brain metastasis |  |
| No | 0 |
| Yes | 1 |

***Abbreviation*:** Mets, metastasis; At DX, at the time of diagnosis; LN, lymph nodes.
