# Supplementary material for: Machine Learning‐Based Prediction of Brain Metastasis at Initial Diagnosis in Small‐Cell Lung Cancer: Model Development and SHAP Interpretation Study
Source: Cancer Rep (Hoboken). 2026 Jul 16;9(7):e70625. doi: 10.1002/cnr2.70625 (PMC13374636; doi:10.1002/cnr2.70625)
Supplement: Supplementary file 4 — Table S3: Performance of XGB model in the external validation set. [file CNR2-9-e70625-s001.docx]

**Supplement Table S3.** Performance of XGB model in the external validation set.

| **Model** | **AUC** | **Accuracy** | **Precision** | **Recall** | **F1-Score** | **MCC** | **Brier Score** | **AUPRC** |
| --- | --- | --- | --- | --- | --- | --- | --- | --- |
| XGB | 0.8169 | 0.8907 | 0.8837 | 0.4419 | 0.5891 | 0.5769 | 0.1005 | 0.6647 |

***Abbreviations****:* XGB, extreme gradient boosting machine;AUC, area under the curve; MCC, matthews correlation coefficient.
